# Scoping review of the utilization of wearable devices in pediatric and young adult oncology

**DOI:** 10.1038/s41746-025-01842-5

**Published:** 2025-07-31

**Authors:** Lane Collier, Sarah L. Grimshaw, Julian Stolper, Elyse Passmore, Gareth Ball, David A. Elliott, Rachel Conyers

**Affiliations:** 1 Cancer Therapies, Murdoch Children’s Research Institute, Flemington Road, Parkville, Victoria, 3052 Australia; 2 Department of Paediatrics, University of Melbourne, Flemington Road, Parkville, Victoria, 3052 Australia; 3 Clinical Sciences, Developmental Imaging, Murdoch Children’s Research Institute, Flemington Road, Parkville, Victoria, 3052 Australia; 4 Gait Analysis Laboratory, The Royal Children’s Hospital, Flemington Road, Parkville, Victoria, 3052 Australia; 5 The Novo Nordisk Foundation Centre for Stem Cell Medicine, reNEW and Murdoch Children’s Research Institute, Flemington Road, Parkville, Victoria, 3052 Australia; 6 Australian Regenerative Medicine Institute, Monash University, Clayton, Victoria, 3800 Australia; 7 Children’s Cancer Centre, The Royal Children’s Hospital, Flemington Road, Parkville, Victoria, 3052 Australia

**Keywords:** Cancer, Health care

## Abstract

This review summarizes the current literature on the use of wearable devices for collecting physiological data in pediatric and young adult (0−25 years) oncology. Searches were conducted in MEDLINE, PubMed and Embase, focusing on pediatric and young adult patients with a cancer diagnosis, and utilizing a wearable device during and/or after treatment. Of the 77 articles that met the inclusion criteria, 61 studies primarily used wearable devices as a tool to monitor physiological changes in an interventional or observational setting. Only 16 studies integrated wearable devices as an active component of the intervention. The most reported wearable device brands were ActiGraph (19, 24.7%), FitBit (14, 18.2%), Ambulatory Monitoring Inc. (11, 14.3%) and Philips Respironics (10, 13%). This scoping review offers valuable insights into the current use of wearable devices in pediatric and young adult (0−25 years) oncology but also reveals notable gaps in the literature.

## Introduction

Wearable devices have rapidly diffused into consumer markets over the last decade, offering a unique opportunity to engage in managing health issues. A wearable device is a portable, non-invasive, body-adhered electronic tool designed to collect, monitor and transmit data related to physiological or behavioral parameters. These devices can monitor a wide range of physiological and activity metrics, including but not limited to, electrocardiograms (ECGs), heart rate, respiratory rate, blood oxygen saturation, and physical activity levels. Due to the capacity for real-time, continuous monitoring of patients, and the possibility of timely intervention to improve health outcomes advancements in wearable technology have generated considerable interest within the healthcare system.

In adult oncology, wearable devices have gained significant traction when used to attain data for prognostication, and rehabilitation planning^1^—which is unsurprising given physical activity is the most readily available and recorded wearable data^2^. A review of wearable devices in adult oncology identified 199 studies which utilized devices for oncology prognostication, treatment monitoring or rehabilitation^3^. However, implementation in clinical application and early detection using continuously monitored data (e.g. blood oxygen saturation, ECGs) is limited. The Apple Heart Study, is the largest wearable device research project to date, led by Apple and Standford, to assess the utility of these data to identify early interventions. Over 400,000 participants across the United States of America were closely monitored for irregular heart rhythms (specifically atrial fibrillation), using the Apple Watch in built optical heart rate sensor^4^. This groundbreaking clinical trial found that the wearable device could not only be used to identify irregular heart rhythms but also distinguished episodes of atrial fibrillation in adult participants^4^. This technology is now readily available to the public, allowing individuals to continuously monitor their heart rhythm in real-time and share this information with the appropriate health professionals.

In contrast, the use of wearable devices in pediatrics and young adults (0–25 years) is limited with most wearable studies using activity trackers only. The gap, and potential for intervention with wearable devices, remains for children with chronic illnesses. Wearable devices are an opportunity for improving the long-term outcomes of children with cancer and has a number of potential clinical applications. Children with cancer experience in the first 12 weeks of treatment (on average) 2.5 adverse drug reactions. The most common are nausea, fatigue, vomiting and myelosuppression requiring blood product transfusions^5^. Wearable technologies in pediatric and young adult oncology have the potential to monitor and manage these common treatment-related side effects by providing continuous physiological data. This can enable timely intervention, improve symptom management, and enhance quality of life.

The aim of this scoping review is to understand how wearable devices are being utilized in pediatric and young adult (0–25 years) oncology and the types of physiological data being collected. Furthermore, we aim to report on any potential benefits or inefficacies of the wearable devices.

## Results

### Study selection and characteristics

**Fig. 1:**
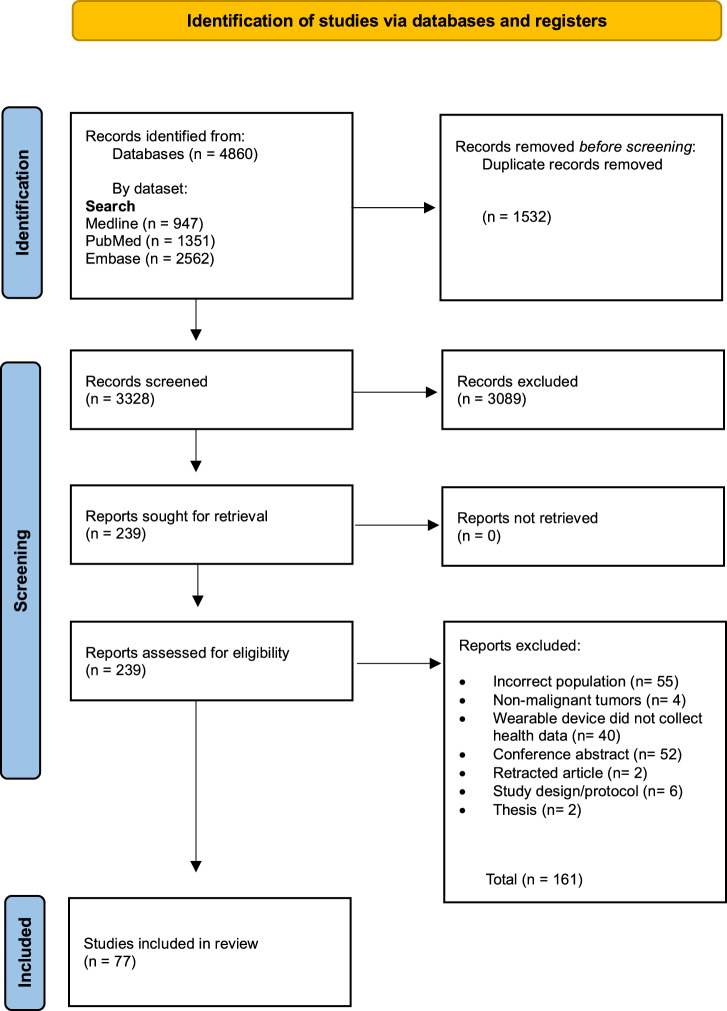
PRISMA flow diagram. The PRISMA flow diagram outlines the scoping review process, including records identified (*n* = 4860), screened (*n* = 3328), and included (*n* = 77) in the final analysis. Diagram created using catchii.org.

The search strategy (see methods) identified 4860 records. After duplicates were removed 3328 articles underwent title and abstract screening by two independent reviewers. Articles that did not meet the inclusion criteria were removed. Secondary selection involved the review of 239 full texts to establish article relevance. The main reasons for article exclusion during the full text review was due to the wearable device not collecting physiological and/or activity data, or the article was a conference abstract. Ultimately, 77 articles were in included in this scoping review^6–82^ (Fig. 1).

Many of the studies were conducted in the United States (USA) (33) or Europe (31), with 6 studies conducted in Australia, 3 in Canada, 2 in Türkiye, 1 in Japan and 1 in Taiwan. As shown in Tables 1, 79.2% of the studies utilized wearable devices primarily as data collection tools, either in observational contexts or to monitor physiological and activity-related outcomes. These applications included tracking physical activity (35.1%), sleep patterns (22.1%), symptoms or treatment-related side effects (15.6%), and other combinations such as physical activity and sleep monitoring (2.6%). In contrast, only 20.8% of the studies utilized the wearable device as part of the intervention itself. Nearly half of the studies (49.3%) were conducted while the patient was at home, 22.1% of the studies were conducted while the patient was in hospital, and 28.6% were conducted both in and out of a clinical setting. Sample sizes ranged from 2−1378 participants (median = 37 participants). Across the included studies, the aims generally fell into three categories: assessing feasibility, monitoring physiological or activity data, and using the device as part of an active intervention. These overarching aims are reflected in how we’ve categorized device use and functionality throughout the results.

**Table 1:** Use and setting of wearable devices across included studies

Characteristic	Subcategory	Description	Number of studies (%)
**Wearable Device Use**	**Data Collection Tool**		**61 (79.2%)**
	Physical Activity Monitoring	Devices measure step count, movement intensity, or activity levels	27 (35.1%)
	Sleep Monitoring	Devices assess sleep duration, quality, or patterns	17 (22.1%)
	Symptom or Toxicity Surveillance	Devices track symptoms or physiological indicators of treatment side effects	12 (15.6%)
	Physical Activity and Sleep Monitoring	Combined monitoring of physical activity and sleep behaviors	2 (2.6%)
	Rehabilitation Support	Devices guide or monitor recovery and physical therapy	2 (2.6%)
	Treatment Monitoring	Devices monitor adherence or response to therapy	1 (1.3%)
	**Intervention Tool**		**16 (20.8%)**
	Intervention	Device functions as an active component of the study intervention	16 (20.8%)
**Study Setting**	Outpatient	Participants monitored in non-hospital/community settings	38 (49.3%)
	Inpatient	Participants monitored in hospital settings	17 (22.1%)
	Mixed	Study includes both inpatient and outpatient populations	22 (28.6%)

This table summarizes how wearable devices were used in the included studies, categorized by purpose, either as a tool for data collection or as an intervention. The lower section details the study settings, including inpatient, outpatient and mixed environments. For a full breakdown of study characteristics refer to Supplementary Table 2.

While gender distribution varied across the 77 studies, females comprised of 50.5% of the overall participant population. 13 studies (16.9%) reported exclusively on patients with acute lymphoblastic leukemia (ALL), 9 studies (11.7%) reported on patients with central nervous system (CNS) tumors and 2 studies (2.6%) reported on patients with bone tumors. 52 studies (67.5%) reported on patients with any cancer diagnosis and 1 study (1.3%) did not state the exact cancer diagnosis of participants. As shown in Table 2, over half (55.8%) of the studies reported on cancer patients currently receiving treatment, 36.4% reported on patients who have completed treatment, and 7.8% of studies included a mixed cohort of patients on and off treatment. A full breakdown of study characteristics is available in Supplementary Table 1.

**Table 2:** Participant demographics

Demographics	Subcategory	Number of Studies (%)
**Cancer Diagnosis**	Any	52 (67.5%)
	ALL	13 (16.9%)
	CNS Tumors	9 (11.7%)
	Bone Tumors	2 (2.6%)
	Unknown	1 (1.3%)
**Stage of Treatment**	On Treatment	43 (55.8%)
	Off Treatment	28 (36.4%)
	Both	6 (7.8%)

This table summarizes the cancer diagnosis and treatment stages of participants across included studies. For full participant demographic details, refer to Supplementary Table 1.

### Wearable devices

The most reported brands of wearable devices were ActiGraph (19 studies, 24.7%), FitBit (14 studies, 18.2%), Ambulatory Monitoring Inc (11 studies, 14.3%) and Philips Respironics (10 studies, 13%). An accelerometer was the most common sensor used across 70% of the studies. Two studies connected one wearable device to another, to collect physiological information which would in turn influence the second device^63,64^. Table 3 lists all wearable device brands utilized across the 77 studies and their associated sensors. While many wearable device brands (e.g., Fitbit, Biofourmis) can measure multiple physiological parameters through various integrated sensors, we only report on the specific sensor modalities explicitly described within the included studies (*n* = 77). For full details on the devices and functionality utilized for each individual study, please refer to Supplementary Table 2.

**Table 3:** Wearable device brand and sensors

Brand	Sensor type(s) used in studies	Number of studies (%)
ActiGraph	Accelerometer	19 (24.7%)
Fitbit	Accelerometer, Optical Heart Rate Sensor, Blood Oxygen Sensor (some models)	14 (18.2%)
Ambulatory Monitoring, Inc.	Accelerometer, Light Sensor,	11 (14.3%)
Philips Respironics	Accelerometer, Light sensor	10 (13%)
Biofourmis	Optical Heart Rate Sensor, Respiratory Rate Sensor, Temperature Sensor, Blood Oxygen Sensor	3 (3.9%)
Oculus/Meta	Activated by Biofeedback loop, Eye movement	3 (3.9%)
Activinsights	Accelerometer	2 (2.6%)
Garmin	Accelerometer, Optical Heart Rate Sensor	2 (2.6%)
Movisens	Accelerometer	2 (2.6%)
Orthocare Innovations	Accelerometer	2 (2.6%)
Axivity	Accelerometer	1 (1.3%)
BlueSpark Technologies	Temperature Sensor	1 (1.3%)
BTL	ECG, Heart Rate Sensor	1 (1.3%)
greenTEG	Temperature Sensor	1 (1.3%)
Modus Health	Accelerometer	1 (1.3%)
SWA BodyMedia	Accelerometer, Temperature Sensor	1 (1.3%)
Unspecified	Various, refer to Supplementary Table 2.	6 (7.8%)

This table summarizes wearable device brands, and the sensor types reported as being used in each study. The listed sensors reflect only those explicitly described in the study methods and may not represent the full range of sensors available in the devices. For detailed device information by study, refer to Supplementary Table 2.

Over half of the studies (42 studies, 55%) used wearable devices that were worn on the wrist/arm, with 18 studies (23.4%) providing participants with hip-worn devices. The remaining 17 studies used devices placed on the patient’s skin, clothes, chest or head. Most of the wearable devices utilized physical activity, step count and sleep detection capabilities (Table 4). In addition, only 11 (14.3%) studies utilized more advanced wearable device capabilities such as respiratory rate, blood oxygen, ECG, heart rhythm and eye movement. While wearable device brands and their primary functionalities are presented in separate tables to preserve accuracy, often studies made use of different functionalities within the same device. For example, Fitbit devices were used to collect step count data in some studies and heart rate data in others, depending on the device model and study aim. Supplementary Table 2 summarize these uses.

**Table 4:** Wearable device functionality

Functionality	Number of study devices (%)
Physical Activity	44 (57.1%)
Steps	33 (42.9%)
Sleep	29 (37.7%)
Heart Rate	6 (7.8%)
Temperature	6 (7.8%)
Respiratory Rate	5 (6.5%)
Blood Oxygen	3 (3.9%)
ECG	1 (1.3%)
Heart Rhythm	1 (1.3%)
Eye Movement	1 (1.3%)
Angular Velocity of the arm	1 (1.3%)

This table summarizes the functionalities of wearable devices as reported in the included studies. Functionalities reflect only those explicitly described as used or analyzed in each study and may not represent all capabilities of the devices. For additional detail, refer to Supplementary Table 2.

The duration of time participants were asked to wear the wearable device ranged from 5 min to 1 year, however, the majority of studies utilized the wearable devices for <1 month. Only twenty-five studies reported on the wear time adherence, with studies reporting high adherence at the start of the study, but adherence decline over time, particularly for longer studies^7,13,17,18,20–23,26,27,36,37,40,41,43,48,58,65,69,75,77–79,81^. Of the studies that reported an average wear time, the overall average was 12.35 h/day. With an overall average wear duration of 5.76 days/week based on studies that reported this data.

Twenty (26%) studies reported patients had technical difficulties with the wearable devices during the study, with the most common problem being data syncing and/or connectivity issues^6,10,19,22,23,25,26,29,32,36,37,40,47,53,57,58,61,78,79,81^. Four studies reported adverse effects as a direct result of the wearable device^22,36,37,47^, with skin irritation being the most common. Detailed data on device placement, functionality, and reported compliance metrics for each study are provided in Supplementary Table 3.

## Discussion

The current scoping review summarizes the literature surrounding the use of wearable devices in pediatric and young adult (0−25 years) oncology, the types of data collected, and the benefits and limitations of the technology. We have identified the use of wearable device is of interest in pediatric and young adult oncology. However, with only 77 studies identified, it has highlighted a clear gap in the literature. In contrast, systematic reviews in adult oncology have identified 199 studies.

Our analysis reveals that the predominant wearable device used in pediatric and young adult oncology is the ActiGraph (25% of studies). The ActiGraph is a wrist-worn accelerometer used to monitor and record physical activity or sleep behavior over time, with a long-standing presence in the wearable device market. ActiGraph is also the predominant device used in adult oncology, used in around 36% of studies^3^. However, within adult oncology there is a wider variety of wearable device brands utilized when compared to pediatric and young adult oncology. In addition, given the size of the wearable device market, and it’s expected revenue of USD 138 billion in 2025^83^, commercial grade devices will become more accessible for oncology research. However, only 19 (25%) studies utilized a commercially available wearable device (e.g. FitBit, Oculus/Meta and Garmin). Of the types of wearable devices used, only Phillips Respironics devices have been validated and approved for use in pediatrics and young adults as a medical device. Other brands are research grade wearable devices. Validation of wearable device data in pediatrics and young adults is technically challenging because of the changes in physiology across the age span from neonates through adolescence. This presents difficulties in interpreting wearable data in real time, particularly where clinical decisions rely on parameters that vary with age, such as heart rate, sleep, and activity patterns^84,85^. For example, distinguishing between sleep and daytime naps in infants and young children may complicate sleep analysis, while physical activity expectations differ greatly between toddlers and adolescents, making standardization complex. The best example of this is heart rate, starting at a median rate of 140 (beats per minute) BPM in neonates, before gradually decreasing during infancy (median 110BPM), early and late childhood (median 100BPM and 90BPM) and adolescence (median 70BPM)^86^. In the context of short-term studies the variability in physiological parameters across the age spectrum can be mitigated by collecting baseline data of participants prior to treatment or intervention. Whilst complex annotation for activity would be required, this could individualize comparisons without the need to monitor across the entire pediatric age range.

Within pediatric and young adult oncology, more research is required in the validation and feasibility of using commercially available products that are readily accessible on the consumer market. Testing of these products within the confines of robust and blinded clinical studies is needed to support the further implementation of wearable devices in this cohort.

Whilst 77 studies used wearable devices for data collection, few monitored emerging functionalities such as heart rhythm monitoring, ECGs, respiratory rate, temperature and blood oxygen. Only 11 studies (14%) utilized these newer functionalities. It is important to note that, given 68% of included studies involved mixed cancer cohorts, it was not possible to draw conclusions about how diagnosis type may influence device functionality or data collection choices. Moreover, very few studies (21%) looked to implement the wearable device as part of an intervention^13,15,30,33,38,40,44,53,63,64,81^. Of these, 7 studies (9%) explicitly stated the utilization of real-time data monitoring (respiratory rate, heart rate, temperature, blood oxygen, activity and steps), with 2 studies (3%) using this real-time respiratory rate data to influence a virtual reality device in a bio-feedback loop^63,64^. In the remaining five studies, real time data was either reviewed by healthcare professionals to inform patient care or used to provide immediate feedback to participants to influence their activity behavior. Despite the limited number of intervention-focused studies, wearable technologies appear to be well-received by patients and survivors, who are increasingly open to integrating these tools into their care experience^87,88^. One retrospective study analyzed electronic medical records to identify how many referrals to a pediatric arrythmia clinic were prompted by identification of abnormal heart rhythms using the Apple Watch device. Interestingly, in 71% of cases (*n* = 145) the Apple Watch electrocardiogram findings prompted the cardiology team to pursue further workup^88^. In addition, health organizations across the United States of America have started to integrate wearable device data directly into patient electronic medical records to utilize real-time data to assist with patient care^89^. These studies show the opportunities for early intervention that are consumer led rather than investigator-initiated trial lead. Pediatric and young adult patients particularly benefit from wearable devices as they enable continuous, non-invasive monitoring that can reduce hospital visits and clinical burden. They also offer the potential for early detection of physiological changes, promote self-management and engagement with their own health, and support tailored interventions that accommodate developmental and behavioral variability unique to children and adolescents^36,40,81^. Furthermore, real-time monitoring and feedback can empower caregivers and clinicians to make timely decisions, potentially improving clinical outcomes and quality of life.

A total of 21 studies included in this scoping review assessed feasibility^6,10,19,22,23,25,26,29,30,32,36,37,40,47,53,58,61,78,79,81,90^, with reported barriers to implementation including well-established issues in patient compliance, technical issues resulting in data failure, and the clinical validation of the device^91,92^. However, it is important to note that compliance in pediatrics is dependent on the parent/guardian and patient. No studies in our scoping review reported on parental compliance of enforcing or facilitating wearable device wear and this remains a research gap. Another factor when considering a wearable device in pediatric and young adult oncology is the placement of the device and compliance with wearing it appropriately. While wrist and hip worn devices commonly use accelerometers to measure physical activity, compliance appears to be lower with hip placement, particularly in younger children^16,31,80^. This suggests that device placement itself, regardless of sensor type, may influence wear compliance due to comfort and acceptability. In this context, three studies reported on the lower compliance in younger children when using hip worn devices but didn’t specify how this differed from older participants. The reported information in a study by Hooke et al. was that the device on the hip make them feel “different”, whilst Wu et al. reported two participants withdrawing from the study due to discomfort of wearing the hip-worn device.

Technical issues were a reported barrier to wearable use in children. 26% of studies reported connectivity and data syncing problems that impacted the quality and/or quantity of data available to researchers (see Supplementary Table 3 for full details). However, many studies did not provide detailed descriptions of these technical issues, limiting insight into their nature and resolution. In the context of the broader literature with respect to wearable technology, reported issues include dropped connections, signal interference, pairing problems, low battery impacting connectivity, incompatible devices, environmental factors and software glitches^93,94^. In assessing wearable devices in sensitive setting such as pediatric and young adult oncology, ease of use and careful study design to avoid these common technical issues is essential. Private healthcare providers with existing medical devices, such as Kaiser Permanente or Ochsner, have addressed these concerns by implementing on-site technology assistance, similar to the Apple ‘Genius Bar’, to troubleshoot any technical issues that may arise^92^. This could be a viable solution to overcoming technical issues but should be explored in both public and private sectors, allowing access for all patients.

While wearable technology holds significant promise in pediatric oncology, health equity must be a central consideration in its development and implementation^87,95^. Access to wearable technology and consistent internet connectivity is not universal and may be limited for families from lower socioeconomic backgrounds, rural areas, or marginalized communities^96–99^. This divide, risks excluding those who may already experience health disparities, thereby widening inequities in care and outcomes. Furthermore, health literacy, language barriers, and varying levels of technological familiarity among caregivers may influence the ability to use these tools effectively^100^. To mitigate this, future studies and programs should incorporate equity-focused strategies, such as, providing devices and/or data plans, offering multilingual support, and involving diverse communities in study design. This will ensure that the benefits of wearable technologies are accessible to all pediatric and young adult cancer patients.

Although 28 studies (36%) reported on the acceptability and ease of use of the wearable devices^9,16,19,22,25,29–33,36,37,40,41,44,47,48,53,56,58,61,64,69,73,78,80–82^, none of them reported on the thoughts, attitudes and barriers identified from consumers (defined here as pediatrics and young adults and/or their caregivers) around using the wearable device to detect diseases or treatment adverse reactions. This is likely due to minimal studies exploring the wearable device as an independent, stand-alone intervention. This highlights a current gap in the literature which indicates another potential barrier to implementation. While we acknowledge that younger pediatric patients may face developmental or communication-related challenges in articulating their experiences, it remains essential to incorporate their perspectives using age-appropriate and child-centered methods. At the same time, caregiver insights can provide valuable complementary context to better understand attitudes, barriers, and acceptance of wearable devices for treatment-related monitoring.

This scoping review offers valuable insights into the current use of wearable devices in pediatric and young adult oncology but also reveals notable gaps in the literature, particularly when compared to the substantial body of research in adult oncology. Barriers to implementation is an ongoing challenge within this area of research and needs to be explored further in the context of wearable devices being an independent intervention. This scoping review also highlights the need for more validation studies using consumer grade devices, which may make way for more accessible and viable option for research use. Moreover, modern consumer grade devices provide a unique opportunity for a rich dataset of continuous and real time data from patients, without interrupting day to day life and burdening pediatric and young adult patients^19,36,44,81^.

## Methods

This review was conducted according to the guidelines for Preferred Reporting Items for Systematic Reviews and Meta-Analysis Statement – Scoping Review (PRISMA-ScR) and registered with the OSF Registry (osf.io/fsdb9). Completed PRISMA-ScR Checklist is provided in Supplementary Note 1.

### Inclusion and exclusion criteria

Studies were eligible for inclusion if they involved:

- Pediatric, adolescent and young adults aged between 0 and 25 years AND
- Participants had a cancer diagnosis or had received a hematopoietic stem cell transplant AND
- Included wearable devices collecting physiological and/or activity data during and/or after cancer treatment AND
- Articles published in English, full text (or have a translated version available) from 2014 onwards.

Studies were excluded if they involved:

- Pre-cancerous conditions
- Invasive devices (e.g.: insulin pumps)
- Books, chapters, conferences, editorials, comments, theses, protocols, systematic reviews and guidelines

If articles included participants both inside and outside this age range, the results needed to be clearly defined for the pediatric, adolescent and young adult cohorts, or report a median or mean age within the 0–25 year range.

Wearable technology included non-invasive devices e.g. virtual reality devices: and excluded current medical technologies such as hearing aids.

### Search methods

Literature searches were conducted in June 2024 in the following electronic databases; Medline, Embase and PubMed, and restricted to the last 10 years. Medline was used as the primary database for this review. The database selection was developed in consultation with an academic librarian and focused on high-yield, subject-specific sources to ensure relevance and feasibility within the scope of the review.

The search strategy consisted of a combination of Medical exploded Subject Headings (MeSH) and various keywords to identify the literature. MeSH terms applied in the database searches included: “Wearable Technology”, “Neoplasms”, “Stem Cell Translation”, “Pediatrics”, “Adolescents”, “Young Adults”. These terms were combined in their associated cluster groups with all word variations included in the search. The search strategy was adjusted to focus on pediatric, adolescent and young adults, with search terms removed if they were not appropriate for this search.

To refine the search, the “AND” operator was applied to combine all distinct concepts and yield relevant results. This approach was consistently applied across all electronic databases used for this review, with adjustments tailored to each database’s subject thesaurus. An additional search was completed on reference lists of similar review articles to ensure all relevant literature was captured. A detailed search has been provided in Supplementary Note 2.

### Article appraisal

Results were uploaded to the systematic review software Catchii. Catchii is a new web-based systematic review platform that streamlines the development of systematic reviews with a user-friendly interface on both computers and mobile devices^101^.

Catchii removed all duplicate articles before two reviewers independently reviewed title and abstracts against the predefined inclusion and exclusion criteria. Any discrepancies were resolved through collaborative discussion, prior to reviewers proceeding to screen the full text articles. Following the two-stage screening process, a further collaborative discussion was undertaken to reach consensus. One reviewer extracted the data from Catchii into an excel spreadsheet consisting of:

- Study title
- Author(s)
- Year published
- Country of study
- Setting (home/hospital/both)
- Data collection tool or Intervention (Wearable Device)
- Study design
- Study primary aim(s)
- Stage of treatment (on/off/mixed)
- Number of participants
- Age of participants
- Gender of participants
- Diagnosis of participants
- Wearable device brand
- Wearable device name
- Where wearable device was worn during study
- Expected wear time of wearable device
- Minimum wear time of wearable device for valid data?
- How is wear time calculated?
- Actual wear time
- Sampling frequency of wearable device
- Physiological and/or activity data collected via the wearable device
- Benefits of the wearable device
- Inefficacies of the wearable device

A second reviewer independently checked the entered data.

### Data analysis

The primary outcome of this scoping review was the use of wearable devices in pediatric oncology, with the secondary outcome being its benefits or limitations. Data were analyzed using descriptive statistics to identify common themes related to these outcomes.

## Supplementary information

Supplementary Material

## Data Availability

The data generated and analyzed during this study are largely available within the manuscript and supplementary material. Any additional data supporting the findings of this study can be made available upon reasonable request to the corresponding author.

## Code availability

This study did not involve the utilization of any custom code or algorithm.
