# Supplementary material for: Scoping review of the utilization of wearable devices in pediatric and young adult oncology
Source: NPJ Digit Med. 2025 Jul 31;8:488. doi: 10.1038/s41746-025-01842-5 (PMC12313918; doi:10.1038/s41746-025-01842-5)
Supplement: Supplementary file 1 — Supplementary Material [file 41746_2025_1842_MOESM1_ESM.pdf]

Supplementary Table 1: Detailed characteristics of included studies and participants.

Ref	Country	Total sample size	Sex (%F)	Age (Mean/Median)	Age Spread (SD/IQR/Range)	Cancer Diagnosis	Treatment Status	Study Location
**Wearable Device Use: Data Collection Tool (n=61)**								
Muller 2016	DE	150	48.7%	M 10.7	SD 4.3	57.4% Leukemia/Lymphoma<br>25.3% CNS/Neuro-oncology<br>17.3% Sarcoma	Off	Mixed
Rosen 2015	US	50	56%	Mdn 4.5	NR	100% Leukemia/Lymphoma	On	Mixed
Muller 2014	DE	21	**Control** 54.5%<br>**Intervention** 60%	**Control** M 12.2<br>**Intervention** M 15.2	NR	100% Sarcoma	On	Mixed
Rogers 2014	US	87	34.5%	M 8.8	SD 3.3	100% Leukemia/Lymphoma	On	Outpatient

Marmol-Perez 2024	ES	116	42.2%	M 12.1	SD 3.3	50.9% Leukemia/Lymphoma<br><br>16.4% CNS/Neuro-oncology<br><br>15.4% Solid Tumors<br><br>11.2% Sarcoma<br><br>5.2% Histiocytosis<br><br>0.9% Unknown	Off	Outpatient
Bratteteig 2024	NO<br>CH	270	**Control**<br>50%<br><br>**Intervention**<br>47%	**Control**<br>M 13.2<br><br>**Intervention**<br>M 13.4	**Control**<br>SD 2.6<br><br>**Intervention**<br>SD 2.5	**Control**<br>Healthy participants<br><br>**Intervention**<br>59.9% Leukemia/Lymphoma<br><br>28.7% Other<br><br>11.5% CNS/Neuro-oncology	Off	Outpatient
Ha 2023	AU	37	54.1%	M 11.7	SD 3	81.1% Leukemia/Lymphoma<br><br>21.6% Other<br><br>13.5% CNS/Neuro-oncology	Off	Outpatient

Grydeland 2023	NO<br>DE<br>DK<br>FI<br>CH	432	47.7%	M 12.2	SD 2.2	57.9% Leukemia/Lymphoma<br>17.8% Other<br>16% CNS/Neuro-oncology<br>8.3% Sarcoma	Off	Outpatient
Hooke 2023	US	15	73.3%	M 10.3	SD 4.5	100% Leukemia/Lymphoma	On	Mixed
Gaser 2022	DE	41	34.1%	M 10	SD 4	100% Leukemia/Lymphoma	On	Mixed
Rehorst-Kleinlugtenbelt 2019	NL	25	36%	Mdn 8.2	NR	68% Leukemia/Lymphoma<br>32% Solid Tumors	On	Mixed
Nunes 2019	BR<br>PT<br>US	118	66.1%	NR	R 8-18	56.8% Leukemia/Lymphoma<br>23.7% Other<br>19.5% Sarcoma	On	Inpatient
Johnson 2018	US	21	47.6%	NR	R 8-12	100% CNS/Neuro-oncology	Off	Outpatient

Darezzo 2015	US	35	51.4%	NR	R 8-17	42.9% Leukemia/Lymphoma<br>31.4% Sarcoma<br>25.7% Other	On	Outpatient
Van Deuren 2020	NL	231	**CFS**<br>73.5%<br>**ACS**<br>50.5%<br>**CCS**<br>73.5%	**CFS**<br>M 24.19<br>**ACS**<br>M 45.60<br>**CCS**<br>M 23.27	**CFS**<br>SD 6.27<br>**ACS**<br>SD 9.46<br>**CCS**<br>SD 6.97	**CFS**<br>Chronic Fatigue Syndrome<br>**ACS**<br>71.6% Solid Tumors<br>17.9% Leukemia/Lymphoma<br>7.4% Other<br>3.2% Sarcoma<br>**CCS**<br>58.8% Leukemia/Lymphoma<br>17.6% Sarcoma<br>11.8% Solid Tumors<br>8.8% CNS/Neuro-oncology<br>2.9% Other	Off	Outpatient

Traube 2020	US	56	46.4%	NR	R 0-18	26.8% Other<br><br>25% Sarcoma<br><br>32.1% CNS/Neuro-oncology<br><br>16.1% Leukemia/Lymphoma	On	Inpatient
Hooke 2019	US	57	**Control**<br>48%<br><br>**Intervention**<br>37%	**Control**<br>M 12.8<br><br>**Intervention**<br>M 12	**Control**<br>SD 3.3<br><br>**Intervention**<br>SD 3.6	**Control**<br>63% Leukemia/Lymphoma<br><br>37% Solid Tumors<br><br>**Intervention**<br>62.5% Leukemia/Lymphoma<br><br>37.5% Solid Tumors	On	Mixed
Matthews 2014	US	52	NR	**Control**<br>M 6.2<br><br>**Intervention**<br>M 6.4	NR	**Control**<br>Healthy Participants<br><br>**Intervention**<br>100% Leukemia/Lymphoma	On	Outpatient
Nessle 2022	US	2	50%	NR	1 & 17 (n=2)	100% Leukemia/Lymphoma	On	Mixed

Fiuza-Luces 2017	ES	49	**Control**<br>28%<br><br>**Intervention**<br>29.2%	**Control**<br>M 11<br><br>**Intervention**<br>M 10	**Control**<br>SD 1<br><br>**Intervention**<br>SD 1	**Control**<br>48% Sarcoma<br><br>20% Leukemia/Lymphoma<br><br>20% CNS/Neuro-oncology<br><br>4% Solid Tumors<br><br>**Intervention**<br>54% Leukemia/Lymphoma<br><br>50% Sarcoma<br><br>8% CNS/Neuro-oncology	On	Inpatient
Grimshaw 2024	AU	20	45%	Mdn 13	NR	90% Leukemia/Lymphoma<br><br>5% CNS/Neuro-oncology<br><br>5% Sarcoma	On	Inpatient
Pitt 2023	AUS	30	56.7%	M 22.2	NR	76.7% Leukemia/Lymphoma<br><br>13.3% Solid Tumors<br><br>10% CNS/Neuro-oncology	Off	Outpatient

Williamson 2023	US	44	52.3%	M15.1	SD 1.2	54.5% Leukemia/Lymphoma<br><br>41% Solid Tumors<br><br>4.5% Other Non-Malignant	Off	Outpatient
Hoag 2022	US	49	**Control**<br>48.3%<br><br>**Intervention**<br>45%	**Control**<br>Mdn 13<br><br>**Intervention**<br>Mdn 12	**Control**<br>IQR 11-15<br><br>**Intervention**<br>IQR 10.5-15	**Control**<br>55.2% Leukemia/lymphoma<br><br>20.7% Other Non-malignant<br><br>17.2% Solid Tumors<br><br>6.9% CNS/Neuro-oncology<br><br>**Intervention**<br>70% Leukemia/Lymphoma<br><br>15% Solid Tumors<br><br>10% Other Non-malignant<br><br>5% CNS/Neuro-oncology	On	Inpatient
Koenig 2024	CH	20	35%	Mdn 8	NR	60% Leukemia/Lymphoma<br><br>25% Solid Tumors<br><br>15% CNS/Neuro-oncology	On	Mixed

Swartz 2020	US	80	46.3%	M 18.1	SD 7.5	38.8% Sarcoma<br>37.6% Leukemia/Lymphoma<br>16.3% Other<br>5% CNS/Neuro-oncology<br>2.5% Solid Tumors	Mixed	Mixed
Dalla 2023	AU	14	28.7%	M 8	NR	50% Leukemia/Lymphoma<br>50% Solid Tumors	On	Inpatient
DeNysschen 2021	US	24	41.7%	M 16.6	SD 2.8	NR	Off	Outpatient
Wang 2024	US	13	53.8%	Mdn 13.2	NR	61.5% Malignancies<br>23.1% Bone Marrow Failure<br>7.7% Hemoglobinopathy<br>7.7% Autoimmune Disease	On	Inpatient
Lazar 2023	RO	35	48.6%	M 5.97	SD 3.76	100% Leukemia/Lymphoma	On	Inpatient

Vyhlidal 2022	CZ	64	**Control**<br>68.4%<br><br>**Intervention**<br>53.8%	NR	R 7-15	**Control**<br>Healthy participants<br><br>**Intervention**<br>53.8% Leukemia/Lymphoma<br><br>46.2% Solid Tumors	Off	Inpatient
Bratteteig 2022	NO<br>CH	157	46.5%	M 13.4	SD 2.5	59.9% Leukemia/Lymphoma<br><br>28.7% Other<br><br>11.5% CNS/Neuro-oncology	Off	Outpatient
Gaser 2022	DE	41	**Control**<br>40%<br><br>**Intervention**<br>28.6%	**Control**<br>M 9.7<br><br>**Intervention**<br>M 10.2	**Control**<br>SD 3.9<br><br>**Intervention**<br>SD 4.2	100% Leukemia/Lymphoma	On	Mixed
Vyhlidal 2022	CZ	26	53.8%	NR	R 7-15	53.8% Leukemia/Lymphoma<br><br>46.2% Solid Tumors	Off	Outpatient
Steur 2020	NL	126	40.50%	Mdn 5.1	IQR 3.1-9.3	100% ALL	On	Outpatient

Pickering 2021	DK	68	38.2%	**CRST**<br>M 12.2<br><br>**Non-CRST**<br>M 11.9	NR	100% CNS/Neuro-oncology	Off	Outpatient
Rogers 2020	US	59	**Control**<br>66.7%<br><br>**Intervention**<br>55.2%`	**Control**<br>M 15.5<br><br>**Intervention**<br>M 16.2	**Control**<br>SD 1.6<br><br>**Intervention**<br>SD 1.6	**Control**<br>Healthy participants<br><br>**Intervention**<br>82.8% Leukemia/Lymphoma<br><br>10.3% Sarcoma<br><br>6.9% CNS/Neuro-oncology	Off	Outpatient
Orsey 2016	US	18	16.7%	NR	R 8-18	77.8% Leukemia/Lymphoma<br><br>16.7% CNS/Neuro-oncology<br><br>5.6% Solid Tumors	On	Outpatient
Setoyama 2016	JP	11	63.6%	M 5.5	NR	100% Leukemia/Lymphoma	On	Mixed

Jacobs 2016	US	34	**Control**<br>33%<br><br>**Intervention**<br>25%	**Control**<br>M 16<br><br>**Intervention**<br>M 15.5	**Control**<br>SD 2.5<br><br>**Intervention**<br>SD 2.6	**Control**<br>66.7% Leukemia/Lymphoma<br><br>22.2 Sarcoma<br><br>11.1% CNS/Neuro-oncology<br><br>**Intervention**<br>81.3% Leukemia/Lymphoma<br><br>18.7% Sarcoma	On	Inpatient
Daniel 2024	US	50	48%	M 14.06	SD 3.58	56% Leukemia/Lymphoma<br><br>32% Other Non-Malignant<br><br>8% Solid Tumors<br><br>2% CNS/Neuro-oncology<br><br>2% Other Malignant	On	Inpatient
Vyhlidal 2023	CZ	41	**Control**<br>62%<br><br>**Intervention**<br>45%	Mdn 24.2	IQR 5.4	**Control**<br>Healthy participants<br><br>**Intervention**<br>100% Leukemia/Lymphoma	Off	Outpatient

Merz 2023	CA	79	45.8%	**Control**<br>M 11.12<br><br>**SSC**<br>M 12<br><br>**CS**<br>M 10.97<br><br>**Intervention**<br>M 11.13	**Control**<br>SD 2.34<br><br>**SSC**<br>SD 3.54<br><br>**CS**<br>SD 2.35<br><br>**Intervention**<br>SD 2.53	**Control/SSC/CS**<br>Healthy participants<br><br>**Intervention**<br>100% Leukemia/Lymphoma	Off	Outpatient
Wu 2023	TW	32	40.6%	Mdn 15	IQR 3.75	68.8% Leukemia/Lymphoma<br><br>31.2% Other Malignancies	On	Mixed
Van Hulst 2023	NL	52	**Control**<br>38.5%<br><br>**Intervention**<br>38.5%	Mdn 5.5	IQR 3-18.8	100% Leukemia/Lymphoma	On	Inpatient
Withycombe 2022	US	65	52.3%	M 13.4	SD 2.8	58.5% Leukemia/Lymphoma<br><br>24.6% Solid Tumors<br><br>16.9% CNS/Neuro-oncology	On	Mixed

Gotte 2023	DE	1378	**Control**<br>52.3%<br><br>**Intervention**<br>50%	**Control**<br>NR<br><br>**Intervention**<br>M 12.3	**Control**<br>R 9-15<br><br>**Intervention**<br>SD 1.9	**Control**<br>Healthy participants<br><br>**Intervention**<br>46% Leukemia/Lymphoma<br><br>32.4% Other<br><br>21.6% CNS/Neuro-oncology	Off	Outpatient
Miller 2021	US	19	52.6%	M 19.7	NR	50% Other<br><br>33.4% Leukemia/Lymphoma<br><br>16.7% CNS/Neuro-oncology	Off	Outpatient
Steur 2020	NL	124	39.5%	Mdn 5.1	IQR 3.1-9.2	100% Leukemia/Lymphoma	On	Outpatient
Rogers 2020	US	33	39.4%	M 9.5	SD 3.9	100% CNS/Neuro-oncology	On	Inpatient
Steur 2020	NL	194	**Control**<br>44.2%<br><br>**Intervention**<br>40.70%	**Control**<br>M 4.8<br><br>**Intervention**<br>M 5	NR	100% Leukemia/Lymphoma	On	Outpatient
Rogers 2019	US	33	39.4%	M 9.5	SD 3.9	100% CNS/Neuro-oncology	On	Mixed

Van Dijk-Lokkart 2019	NL	68	47.1%	M 13.2	SD 3.1	67.6% Leukemia/Lymphoma<br><br>22.1% Solid Tumors<br><br>10.3% CNS/Neuro-oncology	Mixed	Outpatient
Hamari 2019	FI	36	**Control**<br>36.3%<br><br>**Intervention**<br>29.4%	**Control**<br>M 7.9<br><br>**Intervention**<br>M 7.8	NR	**Control**<br>76.1% Leukemia/Lymphoma<br><br>14.3% Other<br><br>9.5% Wilms Tumors<br><br>**Intervention**<br>80% ALL<br><br>20% Other Malignant	On	Mixed
Long 2018	AU	13	53.8%	Mdn 19	NR	100% CNS/Neuro-oncology	Off	Outpatient
Graef 2018	US	37	43.2%	M 9.6	SD 4.2	100% CNS/Neuro-oncology	On	Inpatient
Zupanec 2017	CA	20	**Control**<br>11.1%<br><br>**Intervention**<br>9.1%	**Control**<br>M 6.2<br><br>**Intervention**<br>M 6.3	**Control**<br>SD 2<br><br>**Intervention**<br>SD 1.8	100% Leukemia/Lymphoma	On	Outpatient

Gundle 2017	US	25	56%	M 16	SD 3.3	100% Sarcoma	Mixed	Mixed
Gotte 2017	DE	28	42.9%	M 13.8	SD 2.8	48.6% Sarcoma<br>40.5% Leukemia/Lymphoma<br>10.8% Solid Tumors	On	Mixed
Sabel 2016	SE	13	**Control** 50%<br>**Intervention** 57.10%	**Control** M 13.2<br>**Intervention** M11.9	**Control** SD 1.9<br>**Intervention** SD 3.6	100% CNS/Neuro-Oncology	Off	Outpatient
Braam 2016	NL	60	41.7%	M 13.8	NR	64.9% Leukemia/Lymphoma<br>13.3% CNS/Neuro-oncology<br>16.7% Sarcoma<br>5% Other	Off	Outpatient
**Wearable Device Use: Investigational Tool (n=16)**								
Misawa 2024	CA	10	30%	NR	R 10-19	100% CNS/Neuro-oncology	Mixed	Outpatient

Savas 2024	TR	15	46.7%	M 9.66	SD 2.10	73.3% Leukemia/Lymphoma<br><br>20% CNS/Neuro-oncology<br><br>6.7% Sarcoma	On	Inpatient
Savas 2024	TR	62	**Control**<br>51.6%<br><br>**Intervention**<br>45.2%	**Control**<br>M 9.74<br><br>**Intervention**<br>M 9.33	**Control**<br>SD 1.76<br><br>**Intervention**<br>SD 2.08	**Control**<br>74.3% Leukemia/Lymphoma<br><br>22.6% Sarcoma<br><br>3.2% CNS/Neuro-oncology<br><br>**Intervention**<br>64.5% Leukemia/Lymphoma<br><br>22.6% Sarcoma<br><br>12.9% CNS/Neuro-oncology	On	Inpatient
Ovans 2018	US	15	33.3%	M 11.47	SD 3.33	100% CNS/Neuro-oncology	Mixed	Outpatient
Ha 2022	AU	27	44.4%	M 10	SD 1.5	66.7% Leukemia/Lymphoma<br><br>18.5% Other<br><br>14.8% CNS/Neuro-oncology	Mixed	Outpatient

Krnavek 2021	US	64	**Control**<br>62.5%<br><br>**Intervention**<br>43.8%	**Control**<br>M 22.8<br><br>**Intervention**<br>M 20.5	NR	**Control**<br>Healthy participants<br><br>**Intervention**<br>68.8% Leukemia/Lymphoma<br><br>15.6% Solid Tumors<br><br>9.4% CNS/Neuro-oncology<br><br>6.3% Sarcoma	Off	Inpatient
Gotte 2018	DE	40	**Control**<br>52.6%<br><br>**Intervention**<br>38.1%	**Control**<br>M 15.4<br><br>**Intervention**<br>M 14.5	**Control**<br>SD 3.7<br><br>**Intervention**<br>SD 3.9	**Control**<br>60% Leukemia/Lymphoma<br><br>40% Sarcoma<br><br>**Intervention**<br>80% Leukemia/Lymphoma<br><br>20% Sarcoma	On	Outpatient
Haemmerli 2022	CH	20	45%	M 6	NR	60% Leukemia/Lymphoma<br><br>15% CNS/Neuro-oncology<br><br>15% Solid Tumors<br><br>10% Other	On	Mixed

Koenig 2021	CH	20	45%	Mdn 6	NR	60% Leukemia/Lymphoma<br><br>15% CNS/Neuro-oncology<br><br>15% Solid Tumors<br><br>10% Other	On	Mixed
Devine 2020	US	49	**Control**<br>37.5%<br><br>**Intervention**<br>60%	**Control**<br>M 18.25<br><br>**Intervention**<br>M 18.76	**Control**<br>SD 3.6<br><br>**Intervention**<br>SD 3.9	**Control**<br>87.5% Leukemia/Lymphoma<br><br>12.5% Solid Tumors<br><br>**Intervention**<br>76% Leukemia/Lymphoma<br><br>12% CNS/Neuro-oncology<br><br>12% Solid Tumors	Off	Outpatient
Yurkiewicz 2018	US	33	57.6%	M 22	NR	63.7% Leukemia/Lymphoma<br><br>21.2% Sarcoma<br><br>15.2% Other	On	Mixed

Mendoza 2017	US	59	**Control**<br>60%<br><br>**Intervention**<br>58.60%	**Control**<br>M 16.3<br><br>**Intervention**<br>M 16.9	**Control**<br>SD 1.5<br><br>**Intervention**<br>SD 1.5	**Control**<br>48.3% Solid Tumors<br>44.8% Leukemia/Lymphoma<br>6.9% CNS/Neuro-oncology<br>**Intervention**<br>40% Leukemia/Lymphoma<br>30% Solid Tumors<br>16.7% Sarcoma<br>10% CNS/Neuro-oncology<br>3.3% Other	Off	Outpatient
Le 2017	US	19	73.7%	M 24.3	SD 5.8	63.2% Leukemia<br>26.3% Sarcoma<br>5.3% CNS/Neuro-oncology<br>5.3% Solid Tumors	Off	Outpatient
Hooke 2016	US	16	68.8%	M 8.69	SD 3.09	100% Leukemia/Lymphoma	On	Mixed

Fuemmeler 2020	US	15	40%	M 14.8	SD 1.97	100% Leukemia/Lymphoma	Off	Outpatient
Howell 2018	US	78	**Control**<br>56%<br><br>**Intervention**<br>54.7%	**Control**<br>M 12.4<br><br>**Intervention**<br>M 12.8	NR	**Control**<br>41.7% Leukemia/Lymphoma<br><br>25% CNS/Neuro-oncology<br><br>16.6% Solid Tumors<br><br>12.5% Other<br><br>4.2% Sarcoma<br><br>**Intervention**<br>31.5% Leukemia/Lymphoma<br><br>31.4% CNS/Neuro-oncology<br><br>26% Solid Tumor<br><br>9.3% Sarcoma<br><br>1.9% Other	Off	Outpatient

Supplementary Table 1: Detailed characteristics of included studies and participants.
Countries – AU, Australia; BR, Brazil; CA, Canada; CH, Switzerland; CZ, Czech Republic; DE, Germany; DK, Denmark; ES, Spain; FI, Finland; JP, Japan; NL, Netherlands; NO, Norway; PT, Portugal; RO, Romania; SE, Sweden; TW, Taiwan; US, United States.
Statistical Terms – IQR, interquartile range; M, mean; Mdn, median; NR, not reported; R, range; SD, standard deviation.

Clinical/Study Groups – ACS, adult cancer survivors; CFS, chronic fatigue syndrome; CCS, childhood cancer survivors; CRST, circadian regulatory system tumors; Non-CRST, non-circadian regulatory system tumors; CS, control sibling; SSC, survivor sibling control.

Diagnostic categories were grouped as follows:
Leukemia/Lymphoma – Includes acute lymphoblastic leukemia (ALL), acute myeloid leukemia (AML), Burkitt lymphoma, Hodgkin and non-Hodgkin lymphoma, T-lymphoblastic lymphoma, chronic myeloid leukemia (CML), diffuse large B-cell lymphoma, and general references to leukemia/lymphoma or hematological malignancies.
CNS/Neuro-Oncology – Includes CNS tumors, brain tumors, medulloblastoma, neuroblastoma, ganglioneuroblastoma, germinoma, non-germinoma, and circadian-related brain tumors.
Sarcoma – Includes Ewing sarcoma, osteosarcoma, rhabdomyosarcoma, non-rhabdomyosarcoma, soft tissue sarcomas, and bone tumors.
Solid Tumors – Includes Wilms tumor, retinoblastoma, germ cell tumors, hepatic tumors, testicular cancer, breast cancer, ovarian cancer, malignant epithelial tumors, and other malignant epithelial neoplasms.
Other Malignant – Diagnoses listed as “other” in the original source.
Unknown – Diagnoses listed as “unknown” in the original source.
Non-Malignant – Includes aplastic anemia, sickle cell disease, bone marrow failure, hemoglobinopathies, autoimmune disease, histiocytosis, other non-malignant diagnoses, and unspecified non-malignant conditions.

Supplementary Table 2: Detailed description of wearable device.

Ref	Wearable Device Use Subcategory	Device Brand	Device Name	Where is the device worn?	Minimum wear time	Mean (SD) Actual wear time	Physiological/ Activity Data collected
**Wearable Device Use: Data Collection Tool (n=61)**							
Muller 2016	Rehabilitation	Modus Health	StepWatch 3 Activity Monitor	Ankle	7 days - 8 hours/day	Mean 6.2-6.5 days for 11.6 - 12.2 hours/day	Activity Steps
Rosen 2015	Symptom or Toxicity	Ambulatory Monitoring Inc	MicroMini Motionlogger	Wrist	NR	NR	Sleep
Muller 2014	Rehabilitation	Orthocare Innovations	StepWatch 3TM Activity Monitor	Ankle	7 days - 8 hours/day	NR	Activity
Rogers 2014	Treatment	Ambulatory Monitoring Inc	Mini Motionlogger AAM-32	Wrist	72 hours prior AND 72 hours after DEX	NR	Sleep Activity

Marmol-Perez 2024	Physical Activity	ActiGraph	wGT3x-BT	Wrist	1 day<br>16hrs/day	NR	Sleep<br>Activity
Bratteteig 2024	Symptom or Toxicity	ActiGraph	GT3x-BT	Hip	3 days<br>8hrs/day	NR	Activity
Ha 2023	Physical Activity	Activinsights	GeneActiv	Wrist	3 days<br>10 hrs/day	Mean 6.5 days	Activity<br>Steps
Grydeland 2023	Physical Activity	ActiGraph	GT3X+	Hip	1 day<br>8 hrs/day	Mean 5 (1.7) days with 12.9 (1.7) hours/day	Activity
Hooke 2023	Symptom or Toxicity	ActiGraph	GT3X+	Wrist	3 weekdays<br>6hrs/day<br>AND<br>1 weekend day<br>6 hrs/day	NR	Sleep<br>Activity
Gaser 2022	Physical Activity	Movisens	Move 3	Hip	4 days<br>8 hrs/day	NR	Activity<br>Steps
Rehorst-Kleinlugtenbelt 2019	Physical Activity	Philips Respironics	Actical	Hip	4 days<br>8 hrs/day	NR	Activity<br>Steps

Nunes 2019	Sleep	Ambulatory Monitoring Inc	NR	Wrist	NR	NR	Sleep
Johnson 2018	Symptom or Toxicity	NR	Actigraph	Wrist	NR	NR	Sleep
Darezzo 2015	Symptom or Toxicity	NR	NR	Wrist	NR	NR	Sleep
Van Deuren 2020	Symptom or Toxicity	NR	Actometer	Ankle	NR	NR	Activity
Traube 2020	Sleep	Ambulatory Monitoring Inc	Micro Motionlogger	Wrist or ankle	NR	NR	Sleep
Hooke 2019	Physical Activity	ActiGraph	GT3X+	Waist	Every time point for 2 days<br>10 hrs/day	NR	Activity<br>Steps
Matthews 2014	Sleep	Philips Respironics	Actigraph Version 5.71	Wrist	4 days	NR	Sleep
Nessle 2022	Symptom or Toxicity	BlueSpark Technologies	TempTraq WD	Skin	NR	NR	Temperature

Fiuza-Luces 2017	Physical Activity	ActiGraph	GT3X	NR	NR	NR	Activity
Grimshaw 2024	Physical Activity	FitBit	NR	Wrist	8 hours/day	NR	Activity<br>Steps
Pitt 2023	Physical Activity and Sleep	Garmin	VivoFit Activity Tracker	NR	NR	NR	Sleep<br>Activity<br>Steps
Williamson Lewis 2023	Physical Activity	FitBit	FitBit Flex Tracker	Wrist	5+ days/week<br>4+ weeks	Mean 55.4 (17.4) days	Sleep<br>Activity<br>Steps<br>Calories burnt
Hoag 2022	Physical Activity	FitBit	FitBit Charge HR	Wrist	80% of 24-hour period	NR	HR<br>Steps
Koenig 2024	Symptom or Toxicity	**Device 1:** greenTEG<br><br>**Device 2:** Biovotion	**Device 1:** CORE<br><br>**Device 2:** Everion VSM-1	Upper Arm or Leg	7+ days<br>18 hrs/day	**Device 1:** Median 10 days<br><br>**Device 2:** Median 3 days	**Device 1:** Temperature<br><br>**Device 2:** HR<br>Respiratory Rate<br>Temperature

Swartz 2020	Physical Activity	FitBit	Alta HR<br>OR<br>Inspire HR<br>OR<br>Fitbit Ace	Wrist	NR	Mean 80 days	HR<br>Activity<br>Steps
Dalla 2023	Physical Activity	FitBit	NR	NR	NR	NR	Activity
DeNysschen 2021	Physical Activity	FitBit	Fitbit Flex	Wrist	No minimum time required	NR	Steps
Wang 2024	Sleep	Ambulatory Monitoring Inc	Motionlogger MicroWatch Actigraph	Wrist	5 nights	Median 22 days	Sleep
Lazar 2023	Symptom or Toxicity	BTL	BTL HOLTER	Chest/hip	24 hours at 2 time points	NR	HR<br>ECG<br>Heart Rhythm
Vyhlidal 2022	Sleep	ActiGraph	wGT3X-BT	Wrist	NR	NR	Sleep
Bratteteig 2022	Symptom or Toxicity	ActiGraph	GT3X-BT	Hip	3+ days - 8hours/day	Mean 13 hours/day for 6 days	Activity<br>Steps
Gaser 2022	Physical Activity	Movisens	Move 3 Accelerometer	NR	4 days<br>8 hrs/day	Mean 5.9 (1) days	Activity<br>Steps

Vyhlidal 2022	Physical Activity	ActiGraph	wGT3X+	Wrist	4 days<br>16+ hrs/day (including 1 weekend day)	NR	Activity<br>Steps
Steur 2020	Sleep	ActiGraph	wGT3X-BT	Wrist	72 hours	NR	Sleep
Pickering 2021	Sleep	Philips Respironics	Actiwatch Spectrum PRO	Wrist	NR	NR	Sleep<br>Activity
Rogers 2020	Sleep	Philips Respironics	AW2 Actigraph	Wrist	72 hours	NR	Activity<br>Steps
Orsey 2016	Physical Activity and Sleep	Philips Respironics	Actiwatch 2	Wrist	NR	NR	Sleep<br>Activity
Setoyama 2016	Sleep	Philips Respironics	Actiwatch 2	Wrist	Limitation of the study - need 5+ days (patient only wore for 3)	NR	Sleep<br>Activity
Jacobs 2016	Sleep	Ambulatory Monitoring Inc	Mini Motionlogger AAM-32	Wrist	NR	NR	Sleep

Daniel 2024	Sleep	Ambulatory Monitoring Inc	Micro Motionlogger	NR	NR	NR	Sleep
Vyhlidal 2023	Physical Activity	Axivity	AX3 Accelerometer	Wrist	4 days (including 1 weekend day) 16hrs/day (8 hours needed to be during waking hours)	Mean 6.8 (1.3) days	Activity Steps
Merz 2023	Sleep	Philips Respironics	Actiwatch 2	Wrist	NR	NR	Sleep
Wu 2023	Physical Activity	ActiGraph	GT3X	Hip	NR	NR	Steps
Van Hulst 2023	Sleep	ActiGraph	wGT3X-BT	Wrist	NR	NR	Sleep
Withycombe 2022	Physical Activity	Garmin	Garmin VivoFit 3	Wrist	4 days at each time point 10 hrs/day	Mean 14.8 hours/day	Steps
Gotte 2023	Physical Activity	ActiGraph	wGT3X-BT	Hip	4 days & 1 weekend day 8hrs/day	Mean 13 hours/day	Activity Steps

Miller 2021	Physical Activity	ActiGraph	GT3x	Hip	3 days & 1 weekend day 6hrs/day	NR	Activity Steps
Steur 2020	Symptom or Toxicity	ActiGraph	wGT3X-BT	Wrist	NR	NR	Sleep Activity
Rogers 2020	Sleep	Ambulatory Monitoring Inc	Micromini Actigraph	Wrist	72 hours	NR	Sleep Activity
Steur 2020	Sleep	ActiGraph	wGT3X-BT	Wrist	72 hours	NR	Sleep
Rogers 2019	Symptom or Toxicity	Ambulatory Monitoring Inc	Micromini Actigraph	Wrist	72 hours	NR	Sleep
Van Dijk-Lokkart 2019	Physical Activity	Philips Respironics	Actical Activity Monitor B Series	Hip	500min/day	NR	Activity Steps
Hamari 2019	Physical Activity	Fitbit	Fitbit Ultra	Waist	8 hours/day	NR	Steps
Long 2018	Physical Activity	Philips Respironics	Actical Accelerometer	Hip	3 days	12+ hours/day (not reported as mean)	Sleep Activity Steps

Graef 2018	Sleep	Ambulatory Monitoring Inc	Micro-mini Motionlogger	Wrist	NR	NR	Sleep
Zupanec 2017	Sleep	Ambulatory Monitoring Inc	Mini Motionlogger	Wrist	Around 3 days/nights	NR	Sleep
Gundle 2017	Physical Activity	FitBit	FitBit one	Waist	4+ days	NR	Activity Steps
Gotte 2017	Physical Activity	Orthocare Innovations	Step Watch 3 Activity Monitor	NR	NR	5.8 (2.8) days, 12.1 (1.5) hours per day	Activity Steps
Sabel 2016	Physical Activity	SWA BodyMedia	SenseWear Pro 2 Armband	Upper Arm	14 hours	NR	Activity Temperature
Braam 2016	Physical Activity	Philips Respironics	Actical MiniMitter	Hip	500min/day	NR	Activity

Wearable Device Use: Investigational Tool (n=16)						
Misawa 2024	Meta Quest	Meta Quest 2/Pro	Headset	NR	NR	Eye Movement<br>Head Movement
Savas 2024	**Device 1:**<br>Oculus<br><br>**Device 2:**<br>NR	**Device 1:**<br>Oculus Quest 2<br><br>**Device 2:**<br>ADXL354	**Device 1:**<br>Headset<br><br>**Device 2:**<br>Chest	NR	NR	**Device 1:**<br>VR device (biofeedback)<br><br>**Device 2:**<br>Respiratory Rate
Savas 2024	**Device 1:**<br>Oculus<br><br>**Device 2:**<br>NR	**Device 1:**<br>Oculus Quest 2<br><br>**Device 2:**<br>ADXL354	**Device 1:**<br>Headset<br><br>**Device 2:**<br>Chest	NR	NR	**Device 1:**<br>VR device (biofeedback)<br><br>**Device 2:**<br>Respiratory Rate
Ovans 2018	FitBit	FitBit Flex Tracker	Wrist	12 weeks	NR	Steps

Ha 2022	Activinsights	GENEActiv	NR	<10 hours/day<br>OR<br>within 1 day there was more than 80% sedentary activity<br>OR<br>valid days was less than 3	NR	Activity
Krnavek 2021	NR	Wrist-worn Frailty Meter	Wrist	5 minutes	NR	Angular velocity of the arm
Gotte 2018	Fitbit	FitBit one or Fitbit Flex	Pocket or wrist	NR	NR	Activity<br>Steps<br>Calories burnt
Haemmerli 2022	Biovotion	Everion VSM-1	Wrist	NR	NR	HR<br>Respiratory Rate<br>Blood Oxygen<br>Temperature
Koenig 2021	Biovotion	Everion VSM-1	Upper Arm or Leg	NR	NR	HR<br>Respiratory Rate<br>Blood Oxygen<br>Temperature

Devine 2020	ActiGraph	wGT3X-BT	Wrist	4 days<br>10+ hrs/day	Mean 6.21 days at baseline. Mean 5.67 days at 3 months	Activity
Yurkiewicz 2018	Fitbit	NR	NR	NR	NR	Sleep<br>Steps
Mendoza 2017	Fitbit	Fitbit Flex	Wrist	4 days<br>8+ hrs/day	NR	Activity<br>Steps<br>Calories burnt
Le 2017	Fitbit	Fitbit One	Waist	NR	Mean 19 (4.7) days/month. Mean 15 (7.9) days/month at 4-6 months	Activity<br>Steps<br>Blood Oxygen
Hooke 2016	Fitbit	FitBit One	Waist	15 days	NR	Activity<br>Steps
Fuemmeler 2020	ActiGraph	GT3X+	Waist	5 days<br>5 hrs/day	NR	Activity<br>Steps
Howell 2018	ActiGraph	wGT3X-BT	Wrist	NR	NR	Activity<br>Steps

Supplementary Table 2: Detailed description of wearable device.
NR, not reported

Supplementary Table 3: Reported benefits or inefficacies of the wearable devices.

Ref	Acceptability	Technical Issues	Adverse Effects
**Wearable Device Use: Data Collection Tool (n=61)**			
Muller 2016	8 patients lost interest in study/wearing device	3 (incomplete data), 2 (lost accelerometer in mail)	NR
Rosen 2015	NR	6 (incorrectly wearing), 1 (lost device)	NR
Muller 2014	NR	NR	NR
Rogers 2014	NR	13 equipment failure or non-compliance	NR
Marmol-Perez 2024	NR	NR	NR
Bratteteig 2024	NR	NR	NR
Ha 2023	NR	Does not record all activity (e.g. swimming)	NR

Grydeland 2023	NR	Accelerometers can't detect all activities (e.g. swimming, cycling or resistance training)	NR
Hooke 2023	Children reported not feeling comfortable wearing the actigraph at school or social settings	Actigraph could not determine all body movements	NR
Gaser 2022	Younger children (ages 4–7) often find hip-worn wearables uncomfortable; wrist-worn sensors are generally better tolerated and more suitable for this age group.	NR	NR
Rehorst-Kleinlugtenbelt 2019	All patients reported the Actical was well-accepted, and the burden and risk was negligible	NR	NR
Nunes 2019	NR	NR	NR
Johnson 2018	NR	NR	NR
Darezzo 2015	NR	NR	NR
Van Deuren 2020	NR	NR	NR
Traube 2020	NR	NR	NR

Hooke 2019	Patients reported discomfort with wearing the actigraph on their waist, expressing that it made them feel 'different' or self-conscious.	NR	NR
Matthews 2014	NR	NR	NR
Nessle 2022	NR	NR	NR
Fiuza-Luces 2017	NR	NR	NR
Grimshaw 2024	Some patients found it uncomfortable to wear when unwell or forgot to charge device	Some patients had issues with syncing the Fitbit	Some issues with security on FitBit dashboard
Pitt 2023	NR	NR	NR
Williamson 2023	Only 30.6% of participants consistently wore the Fitbit throughout the study period. Many participants struggled to remember to wear the device after charging, and some expressed concerns about losing or damaging it. Adolescents were less engaged and accepting of wearable devices. Without incentives, only 30.6% of participants were willing to wear the watch consistently.	Due to issues with the API, data was lost for eight participants. Additionally, four patients did not sync their devices within 30 days, resulting in further data loss. It is essential to establish a reliable data syncing process early in the study to prevent similar issues.	NR

Hoag 2022	Fitbit wristbands not appropriate size for small kids	Fitbit lacks the sensitivity to identify the differences between sleeping and awake	NR
Koenig 2024	The core wearable was worn more frequently than the Everion. Patients considered both devices suitable for temperature recording, with no significant concerns regarding comfort or charging. However, some patients expressed uncertainty when reviewing their data feedback.	Accessing gateway for Everion to allow data transfer was tedious. More technical issues with Everion for data syncing (may be why patients were not as compliant with wearing this device).	4 participants reported occasional sweating under the wearable device leading to irritated skin (3 patients needed some sort of intervention, 1 was fine).
Swartz 2020	Research assistants spent 30% of their time contacting participants to get the FitBit data (team would implement automated reminders next time).	NR	NR
Dalla 2023	Some kids really enjoyed wearing it, others weren't interested. Might depend on age of the child.	NR	NR
DeNysschen 2021	NR	NR	NR
Wang 2024	NR	NR	NR
Lazar 2023	NR	NR	NR

Vyhlidal 2022	NR	NR	NR
Bratteteig 2022	NR	NR	NR
Gaser 2022	NR	NR	NR
Vyhlidal 2022	NR	NR	NR
Steur 2020	NR	NR	NR
Pickering 2021	NR	NR	NR
Rogers 2020	NR	1 patient had technical issues with the actigraph.	NR
Orsey 2016	NR	NR	NR
Setoyama 2016	NR	NR	NR
Jacobs 2016	NR	NR	NR

Daniel 2024	NR	Actigraphy estimates of sleep use algorithms developed in healthy active children, and may overestimate sleep in this largely sedentary hospital setting where patients remain in bed much of the day and night.	NR
Vyhlidal 2023	NR	NR	NR
Merz 2023	NR	NR	NR
Wu 2023	2 patients withdrew from discomfort of wearing actigraph on hip.	NR	NR
Van Hulst 2023	11 participants refused to wear actigraph.	NR	NR
Withycombe 2022	NR	4 patients had accelerometer malfunctions.	NR
Gotte 2023	NR	NR	NR
Miller 2021	NR	NR	NR
Steur 2020	NR	NR	NR
Rogers 2020	NR	NR	NR

Steur 2020	NR	NR	NR
Rogers 2019	NR	NR	No adverse events.
Van Dijk-Lokkart 2019	NR	NR	NR
Hamari 2019	NR	NR	NR
Long 2018	Barriers to implementation was around the intervention activity not the watch.	NR	NR
Graef 2018	NR	NR	NR
Zupanec 2017	Six patients withdrew from the study, with some (exact number unknown) unable to tolerate wearing the device.	NR	NR
Gundle 2017	NR	NR	NR
Gotte 2017	NR	NR	NR
Sabel 2016	NR	NR	NR

Braam 2016	NR	Memory of the wearable device did not allow for 15 second epoch for 7 days. Could only collect for 4 days.	NR
**Wearable Device Use: Investigational Tool (n=16)**			
Misawa 2024	Participants performed 91.2% of the VR intervention.<br>1 patient withdrew (due to time constraints).	No issues with data syncing.	No adverse events related to cybersickness were reported. However, some patients experienced fatigue, dizziness, disorientation, and instability, though none of these symptoms led to study drop-outs.
Savas 2024	The BioVirtualPed relies on Oculus devices, which come with a significant price tag. However, all participants provided positive feedback.	NR	No reported side effects and/or adverse effects.
Savas 2024	NR	NR	NR

Ovans 2018	One patient dropped out before completing week 1, and four others were inconsistent in wearing and using the device. Eleven participants continued through the extended study.	Four patients completed the initial 12 weeks but declined to continue wearing the device for an additional 12 weeks, either because they no longer wanted to wear it or due to frustration with the device not functioning properly.	No adverse effects reported in this cohort.
Ha 2022	59% opt in rate but of the 30 participants who consented, 70% completed the study. 43% reported enjoying the wearable device. Participants would prefer to see their data (unable to with this wearable).	More than half the participants reported at least 1 technical issue. One patient withdrew as a result, other patients resolved the issues with a telehealth call to research team.	No adverse events.
Krnavek 2021	NR	NR	NR
Gotte 2018	94% of patients recommended fitness tracker intervention. 86% said it was easy to use.	Some issues with detecting moderate activity with fitbit.	NR
Haemmerli 2022	NR	NR	NR

Koenig 2021	Most participants considered it a suitable device as it is comfortable to wear and easy to use. A few participants reported problems with the device such as it being too big or difficult to charge.	Wearable device sometimes assigned good quality to an incorrectly measured vital sign. This was most obvious for a 6-year-old child who did not wear the device at all. Though during the first 2 days the device was switched on and recorded pseudo-vital signs in the box, assigning good quality to respiration rate and core temperature.<br>8 participants experienced technically issues e.g. not connecting with app.	Superficial skin lesion (skin healed quickly once removed).<br>Participants reported skin irritation (4), itching (3) and sweating (7).
Devine 2020	NR	NR	NR
Yurkiewicz 2018	85% enjoyed wearing the fitbit, 12% found the fitbit uncomfortable, 15% found it challenging that the watch is not waterproof.	9% had issues with syncing the fitbit to the ipad.	NR
Mendoza 2017	Fitbit was selected for its ease of use. They had no issues recruiting 60 patients. Using a well known/popular device helped with engagement.	NR	NR

Le 2017	4 participants were concerned about the security of the clip-on style fitbit with concerns it would fall off while exercising. All participants would recommend the device, and 20% suggested patients receive it during therapy. Participants suggested another device that is more secure, accurate and waterproof.	5 lost their device, 3 find the device before replacement device was sent and 2 could not find the device and declined a replacement.	NR
Hooke 2016	All participants wore the device with no issue (minus 6-year-old who was removed from the study).	6-year-old patient kept forgetting fitbit at school or attaching it to family dog instead.	NR
Fuemmeler 2020	NR	NR	NR
Howell 2018	16 did not complete the intervention, 8 were lost to follow up, 4 never accessed the website to provide data.	NR	NR

Supplementary Table 3: Reported benefits or inefficacies of the wearable devices.

NR, not reported

Supplementary Note 1: PRISMA-ScR Checklist

Preferred Reporting Items for Systematic reviews and Meta-Analyses extension for Scoping Reviews (PRISMA-ScR) Checklist

SECTION	ITEM	PRISMA-ScR CHECKLIST ITEM	REPORTED ON PAGE #
**TITLE**			
Title	1	Identify the report as a scoping review.	
**ABSTRACT**			
Structured summary	2	Provide a structured summary that includes (as applicable): background, objectives, eligibility criteria, sources of evidence, charting methods, results, and conclusions that relate to the review questions and objectives.	
**INTRODUCTION**			
Rationale	3	Describe the rationale for the review in the context of what is already known. Explain why the review questions/objectives lend themselves to a scoping review approach.	
Objectives	4	Provide an explicit statement of the questions and objectives being addressed with reference to their key elements (e.g., population or participants, concepts, and context) or other relevant key elements used to conceptualize the review questions and/or objectives.	
**METHODS**			
Protocol and registration	5	Indicate whether a review protocol exists; state if and where it can be accessed (e.g., a Web address); and if available, provide registration information, including the registration number.	
Eligibility criteria	6	Specify characteristics of the sources of evidence used as eligibility criteria (e.g., years considered, language, and publication status), and provide a rationale.	
Information sources*	7	Describe all information sources in the search (e.g., databases with dates of coverage and contact with authors to identify additional sources), as well as the date the most recent search was executed.	
Search	8	Present the full electronic search strategy for at least 1 database, including any limits used, such that it could be repeated.	
Selection of sources of evidence†	9	State the process for selecting sources of evidence (i.e., screening and eligibility) included in the scoping review.	
Data charting process‡	10	Describe the methods of charting data from the included sources of evidence (e.g., calibrated forms or forms that have been tested by the team before their use, and whether data charting was done independently or in duplicate) and any processes for obtaining and confirming data from investigators.	
Data items	11	List and define all variables for which data were sought and any assumptions and simplifications made.	
Critical appraisal of individual sources of evidence§	12	If done, provide a rationale for conducting a critical appraisal of included sources of evidence; describe the methods used and how this information was used in any data synthesis (if appropriate).	
Synthesis of results	13	Describe the methods of handling and summarizing the data that were charted.	

SECTION	ITEM	PRISMA-ScR CHECKLIST ITEM	REPORTED ON PAGE #
**RESULTS**			
Selection of sources of evidence	14	Give numbers of sources of evidence screened, assessed for eligibility, and included in the review, with reasons for exclusions at each stage, ideally using a flow diagram.	
Characteristics of sources of evidence	15	For each source of evidence, present characteristics for which data were charted and provide the citations.	
Critical appraisal within sources of evidence	16	If done, present data on critical appraisal of included sources of evidence (see item 12).	
Results of individual sources of evidence	17	For each included source of evidence, present the relevant data that were charted that relate to the review questions and objectives.	
Synthesis of results	18	Summarize and/or present the charting results as they relate to the review questions and objectives.	
**DISCUSSION**			
Summary of evidence	19	Summarize the main results (including an overview of concepts, themes, and types of evidence available), link to the review questions and objectives, and consider the relevance to key groups.	
Limitations	20	Discuss the limitations of the scoping review process.	
Conclusions	21	Provide a general interpretation of the results with respect to the review questions and objectives, as well as potential implications and/or next steps.	
**FUNDING**			
Funding	22	Describe sources of funding for the included sources of evidence, as well as sources of funding for the scoping review. Describe the role of the funders of the scoping review.	

JBI = Joanna Briggs Institute; PRISMA-ScR = Preferred Reporting Items for Systematic reviews and Meta-Analyses extension for Scoping Reviews.

* Where *sources of evidence* (see second footnote) are compiled from, such as bibliographic databases, social media platforms, and Web sites.

† A more inclusive/heterogeneous term used to account for the different types of evidence or data sources (e.g., quantitative and/or qualitative research, expert opinion, and policy documents) that may be eligible in a scoping review as opposed to only studies. This is not to be confused with *information sources* (see first footnote).

‡ The frameworks by Arksey and O'Malley (6) and Levac and colleagues (7) and the JBI guidance (4, 5) refer to the process of data extraction in a scoping review as data charting.

§ The process of systematically examining research evidence to assess its validity, results, and relevance before using it to inform a decision. This term is used for items 12 and 19 instead of "risk of bias" (which is more applicable to systematic reviews of interventions) to include and acknowledge the various sources of evidence that may be used in a scoping review (e.g., quantitative and/or qualitative research, expert opinion, and policy document).

From: Tricco AC, Lillie E, Zarin W, O'Brien KK, Colquhoun H, Levac D, et al. PRISMA Extension for Scoping Reviews (PRISMA-ScR): Checklist and Explanation. Ann Intern Med. ;169:467–473. doi: 10.7326/M18-0850

Supplementary Note 2: Database Search strategy

MEDLINE

1.
exp Neoplasms/

2.
exp Stem Cell Transplantation/

3.
Bone Marrow Transplantation/

4.
(stem-cell-transplant* or bone-marrow-transplant* or hematopoietic or haematopoietic or immunocompromi* or immuno-compromi* or compromised-host* or compromized-host* or immune-compromi* or immunecompromi*).tw,kf.

5.
exp Antineoplastic Agents/ or exp Immunocompromised Host/

6.
drug therapy/ or exp antineoplastic protocols/ or exp chemoprevention/ or exp chemoradiotherapy/ or exp chemotherapy, adjuvant/ or consolidation chemotherapy/

7.
(Adenocarcinoma* or Adeno-carcinoma* or Adenoma* or Angiosarcoma* or Astrocytoma* or Blastoma* or Cancer* or carcinoma* or Carcinosarcoma* or Cholangiocarcinoma* or Chondrosarcoma* or Craniopharyngioma* or Ependymoma* or Ewing* or Ganglioglioma* or Germinoma* or Glioblastoma* or glioma* or Hepatoblastoma* or Hepato-blastoma* or Hepatoma* or Leukaemia* or Leukemia* or Lymphoma* or Malignan* or Medulloblastoma* or Melanoma* or Meningioma* or Mesenchymoma* or Mesothelioma* or Metasta* or

Myeloma* or Neoplas* or Neuroblastoma* or Oncolog* or osteosarcom* or PNET or rhabdomyosarcom* or Retinoblastoma* or Sarcoma* or Seminoma* or Thymoma* or Tumour* or Tumor*).tw,kf.

8.
(chemotherap* or chemo-therap* or antineoplastic* or anti-neoplastic* or chemoprevention* or chemo-prevention* or chemoradiotherap* or chemo-radiotherap* or chemoprophylaxis or chemo-prophylaxis).tw,kf.

9.
1 or 2 or 3 or 4 or 5 or 6 or 7 or 8

10.
exp Wearable Electronic Devices/ or exp virtual reality/ or Virtual Reality Exposure Therapy/

11.
(wearable-device* or wearable-electronic-device* or wearable-e-device* or e-skin or electronic-skin or wearable-technolog* or apple-watch* or fitbit* or biosensing* or bio-sensing* or biosensor* or bio-sensor* or fitness-tracker* or activity-tracker* or head-mounted-display* or head-up-display* or head-worn-display* or google-glass* or smart-glass* or smart-watch* or garmen or galaxy-watch* or wearable-computer* or wearable-sensor* or accelerometer-based-wearable* or virtual-reality or vr or vret).tw,kf.

12.
10 or 11

13.
(newborn* or new-born* or baby or babies or neonat* or neo-nat* or infan* or toddler* or pre-schooler* or preschooler* or kinder or kinders or kindergarten* or kinder-aged or boy or boys or girl or girls or child* or pediatric* or paediatric* or school-age* or schoolage* or schoolchild* or schoolgirl* or schoolboy* or adolescen* or youth or youths or teen or teens or teenage* or juvenile* or minor or minors or underage* or under-age* or school-age* or schoolage* or schoolchild* or schoolgirl* or schoolboy* or young-adult* or young-people* or young-person* or emerging-adult* or emerging-people* or emerging-person* or AYA or AYAs or CAYA or CAYAs).tw,kf,hw.

14.
9 and 12 and 13

Embase

1.
exp neoplasm/

2.
exp stem cell transplantation/

3.
exp bone marrow transplantation/

4.
(stem-cell-transplant* or bone-marrow-transplant* or hematopoietic or haematopoietic or immunocompromi* or immuno-compromi* or compromised-host* or compromized-host* or immune-compromi* or immunecompromi*).tw,kf,dq.

5.
exp antineoplastic agent/

6.
immunocompromised patient/

7.
drug therapy/ or exp chemotherapy/

8.
antineoplastic protocol/

9.
chemoprophylaxis/

10.
exp cancer therapy/

11.
(Adenocarcinoma* or Adeno-carcinoma* or Adenoma* or Angiosarcoma* or Astrocytoma* or Blastoma* or Cancer* or carcinoma* or Carcinosarcoma* or Cholangiocarcinoma* or Chondrosarcoma* or Craniopharyngioma* or Ependymoma* or Ewing* or Ganglioglioma* or Germinoma* or Glioblastoma* or glioma* or Hepatoblastoma* or Hepato-blastoma* or Hepatoma* or Leukaemia* or Leukemia* or Lymphoma* or Malignan* or Medulloblastoma* or Melanoma* or Meningioma* or Mesenchymoma* or Mesothelioma* or Metasta* or Myeloma* or Neoplas* or Neuroblastoma* or Oncolog* or osteosarcom* or PNET or rhabdomyosarcom* or Retinoblastoma* or Sarcoma* or Seminoma* or Thymoma* or Tumour* or Tumor*).tw,kf,dq.

12.
(chemotherap* or chemo-therap* or antineoplastic* or anti-neoplastic* or chemoprevention* or chemo-prevention* or chemoradiotherap* or chemo-radiotherap* or chemoprophylaxis or chemo-prophylaxis).tw,kf,dq.

13.
1 or 2 or 3 or 4 or 5 or 6 or 7 or 8 or 9 or 10 or 11 or 12

14.
wearable device/ or exp wearable computer/ or wearable sensor/ or virtual reality/ or Virtual Reality Exposure Therapy/

15.
(wearable-device* or wearable-electronic-device* or wearable-e-device* or e-skin or electronic-skin or wearable-technolog* or apple-watch* or fitbit* or biosensing* or bio-sensing* or biosensor* or bio-sensor* or fitness-tracker* or activity-tracker* or head-mounted-

display* or head-up-display* or head-worn-display* or google-glass* or smart-glass* or smart-watch* or garmen or galaxy-watch* or wearable-computer* or wearable-sensor* or accelerometer-based-wearable* or virtual-reality or vr or vret).tw,kf,dq.

16.
14 or 15

17.
(newborn* or new-born* or baby or babies or neonat* or neo-nat* or infan* or toddler* or pre-schooler* or preschooler* or kinder or kinders or kindergarten* or kinder-aged or boy or boys or girl or girls or child* or pediatric* or paediatric* or school-age* or schoolage* or schoolchild* or schoolgirl* or schoolboy* or adolescen* or youth or youths or teen or teens or teenage* or juvenile* or minor or minors or underage* or under-age* or school-age* or schoolage* or schoolchild* or schoolgirl* or schoolboy* or young-adult* or young-people* or young-person* or emerging-adult* or emerging-people* or emerging-person* or AYA or AYAs or CAYA or CAYAs).tw,kf,dq.

18.
13 and 16 and 17

PubMed

#1 title/abstract
“stem-cell-transplant*” OR “bone-marrow-transplant*” OR “hematopoietic” OR “haematopoietic” OR “immunocompromi*” OR “immuno-compromi*” OR “compromised-host*” OR “compromized-host*” OR “immune-compromi*” OR “immunecompromi*”

#2 title/abstract
“Adenocarcinoma*” OR “Adeno-carcinoma*” OR “Adenoma*” OR “Angiosarcoma*” OR “Astrocytoma*” OR “Blastoma*” OR “Cancer*” OR “carcinoma*” OR “Carcinosarcoma*” OR “Cholangiocarcinoma*” OR “Chondrosarcoma*” OR “Craniopharyngioma*” OR “Ependymoma*” OR “Ewing*” OR “Ganglioglioma*” OR “Germinoma*” OR “Glioblastoma*” OR “glioma*” OR “Hepatoblastoma*” OR “Hepato-blastoma*” OR “Hepatoma*” OR “Leukaemia*” OR “Leukemia*” OR “Lymphoma*” OR “Malignan*” OR “Medulloblastoma*” OR “Melanoma*” OR “Meningioma*” OR “Mesenchymoma*” OR “Mesothelioma*” OR “Metasta*” OR “Myeloma*” OR “Neoplas*” OR

“Neuroblastoma*” OR “Oncolog*” OR “osteosarcom*” OR “PNET” OR “rhabdomyosarcom*” OR “Retinoblastoma*” OR “Sarcoma*” OR “Seminoma*” OR “Thymoma*” OR “Tumour*” OR “Tumor*”

#3 title/abstract
“chemotherap*” OR “chemo-therap*” OR “antineoplastic*” OR “anti-neoplastic*” OR “chemoprevention*” OR “chemo-prevention*” OR “chemoradiotherap*” OR “chemo-radiotherap*” OR “chemoprophylaxis” OR “chemo-prophylaxis”

#4 #1 OR #2 OR #3

#5 title/abstract
“wearable-device*” OR “wearable-electronic-device*” OR “wearable-e-device*” OR “e-skin” OR “electronic-skin” OR “wearable-technolog*” OR “apple-watch*” OR “fitbit*” OR “biosensing*” OR “bio-sensing*” OR “biosensor*” OR “bio-sensor*” OR “fitness-tracker*” OR “activity-tracker*” OR “head-mounted-display*” OR “head-up-display*” OR “head-worn-display*” OR “google-glass*” OR “smart-glass*” OR “smart-watch*” OR “garmen” OR “galaxy-watch*” OR “wearable-computer*” OR “wearable-sensor*” OR “virtual-reality” OR “vr” OR “vret”

#6 title/abstract
“newborn*” OR “new-born*” OR “baby” OR “babies” OR “neonat*” OR “neo-nat*” OR “infan*” OR “toddler*” OR “pre-schooler*” OR “preschooler*” OR “kinder” OR “kinders” OR “kindergarten*” OR “kinder-aged” OR “boy” OR “boys” OR “girl” OR “girls” OR “child*” OR “pediatric*” OR “paediatric*” OR “school-age*” OR “schoolage*” OR “schoolchild*” OR “schoolgirl*” OR “schoolboy*” OR “adolescen*” OR “youth” OR “youths” OR “teen” OR “teens” OR “teenage*” OR “juvenile*” OR “minor” OR “minors” OR “underage*” OR “under-age*” OR “school-age*” OR “schoolage*” OR “schoolchild*” OR “schoolgirl*” OR “schoolboy*” OR “young-adult*” OR “young-people*” OR “young-person*” OR “emerging-adult*” OR “emerging-people*” OR “emerging-person*” OR “AYA” OR “AYAs” OR “CAYA” OR “CAYAs”

#7 all fields
NOTNLM OR publisher[sb] OR inprocess[sb] OR pubmednotmedline[sb] OR indatareview[sb] OR pubstatusaheadofprint

#8 #4 AND #5 AND #6 AND #7
